# Targeting TREM2 for Parkinson’s Disease: Where to Go?

**DOI:** 10.3389/fimmu.2021.795036

**Published:** 2021-12-24

**Authors:** Xiao-xian Li, Feng Zhang

**Affiliations:** ^1^ Laboratory Animal Center and Key Laboratory of Basic Pharmacology of Ministry of Education, Zunyi Medical University, Zunyi, China; ^2^ Joint International Research Laboratory of Ethnomedicine of Ministry of Education and Key Laboratory of Basic Pharmacology of Guizhou Province, Zunyi Medical University, Zunyi, China

**Keywords:** Parkinson’s disease, pathogenesis, TREM2, immunoregulation, treatment

## Abstract

Parkinson’s disease (PD) is one of most common neurodegenerative disorders caused by a combination of environmental and genetic risk factors. Currently, numerous population genetic studies have shown that polymorphisms in myeloid cell-triggered receptor II (TREM2) are associated with a variety of neurodegenerative disorders. Recently, TREM2 has been verified to represent a promising candidate gene for PD susceptibility and progression. For example, the expression of TREM2 was apparently increased in the prefrontal cortex of PD patients. Moreover, the rare missense mutations in TREM2 (rs75932628, p.R47H) was confirmed to be a risk factor of PD. In addition, overexpression of TREM2 reduced dopaminergic neurodegeneration in the 1-methyl-4-phenyl-1, 2, 3, 6-tetrahydropyridine mouse model of PD. Due to the complex pathogenesis of PD, there is still no effective drug treatment. Thus, TREM2 has received increasing widespread attention as a potential therapeutic target. This review focused on the variation of TREM2 in PD and roles of TREM2 in PD pathogenesis, such as excessive-immune inflammatory response, α-Synuclein aggregation and oxidative stress, to further provide evidence for new immune-related biomarkers and therapies for PD.

## Background

Parkinson’s disease (PD) is the second-largest neurodegenerative disease after Alzheimer’s disease (AD) (1). Amounts of studies have shown immune pathway disorders, including changes in cytokine signals, immune cell proliferation and migration, and phagocytosis are closely involved in dopamine (DA) neurodegeneration (2). Microglia, the main cells in brain immune response, come from primitive hematopoietic progenitors in the yolk sac that serve as the first and most important immune defense line of the central nervous system (CNS) (3). Autopsy analysis, positron emission tomography (PET) imaging and molecular and clinical evidence suggest that microglia activation increases and inflammatory mediators accumulate during DA neurodegeneration (4). Thus, microglia-mediated neuroinflammation plays an important role in the pathogenesis of PD (5).

Myeloid cell-triggered receptor II (TREM2), a class of receptors in the immunoglobulin superfamily, is highly expressed on the surface of microglia in CNS and participates in microglial proliferation, phagocytosis, survival, and expression of inflammatory factors (6–10). There is increasing evidence that TREM2 regulates microglia-mediated neuroinflammation and thus play an important role in neurodegenerative diseases, such as AD, PD and amyotrophic lateral sclerosis (ALS) (11–13). At present, TREM2 is considered to be a risk site for PD, and its genetic variation may increase the risk of PD (14). Thus, understanding of the role of TREM2 in PD and the exploration of its mechanism are conducive to provide new therapeutic targets for PD. This review focused on the variation of TREM2 in PD and roles of TREM2 in PD pathogenesis, such as excessive-immune inflammatory response, α-Synuclein (α-Syn) aggregation and oxidative stress, to further provide evidence for new immune-related biomarkers and therapies for PD.

## Methods: Search Strategy

This review focused on the role of TREM2 on pathogenesis of Parkinson’s disease in human, various animal models and *in vitro* studies. We also summarized the potential regulation of TREM2 on the treatment of Parkinson’s disease. Studies were retrieved from the PubMed and Google Scholar databases using the following search terms: Parkinson’s disease, pathogenesis, TREM2, immunoregulation and treatment. The search terms were applied in different combinations and plural forms, and the search was limited to articles in English. References were screened for additional articles. Retrieval time: from inception to September, 2021.

## PD

PD is characterized by aggregation of α-Syn to form Lewy bodies (LB) and degeneration and loss of DA neurons (15), thus affecting other CNS structures and surrounding tissues. The specific pathogenesis of PD is still unclear. Currently, the pathogenesis of PD is recognized to include excessive-immune inflammatory response, α-Syn aggregation, oxidative stress, mitochondrial dysfunction, endoplasmic reticulum stress, iron-induced neurodegeneration, microbial-gut-brain axis disorder, non-coding RNA regulation, and so on (16–21).

Main motor symptoms of PD are rest tremor, rigidity, bradykinesia and loss of postural reflexes. Non-motor symptoms mainly include cognitive dysfunction, sleep disturbance, anosmia, constipation and depression (22). Moreover, PD patients can be divided into three subtypes: 1) mild motor-predominant; 2) diffuse malignant; 3) intermediate (23). Until now, PD is mostly treated by drug therapy. Deep brain stimulation (DBS) of the subthalamic nucleus and levodopa-carbidopa enteral gel preparation are helpful for advanced-complicated patients or for patients that can be selected on the basis of clinical criteria (24, 25). Also, induced pluripotent stem cells (iPSCs) therapy is one of the potential treatments to slow or even prevent PD progression (26). However, the clinical treatment of PD is symptomatic and cannot control the progression of the disease.

## TREM2

### TREM2 Structure

TREM2 is a cell-surface glycoprotein with an immunoglobulin-like extracellular domain, transmembrane region and short cytosolic tail region, whose single transmembrane helix interacts with DAP12 to mediate downstream signaling, short cytosolic tail terminates signals that lack signal transduction or trafficking motifs (27). The ectodomain of TREM2, which is susceptible to various post-translational modifications and directly interacts with the environment and regulates microglial function, suggesting that the ectodomain is critical for the function of TREM2 itself (28). Homologues sequences located in the ectodomain of TREM2 might be involved in key functions mediated by TREM2 signaling (29). Moreover, the ectodomain of TREM2 is cleaved by a disintegrin and metalloproteinase (ADAM)-10 and ADAM-17 to produce soluble TREM2 (sTREM2), while C-terminal fragment of TREM2 (TREM2-CTF) is cleaved by γ-secretase into intracellular domain (ICD) (30).

### TREM2 Signaling

**Figure 1:**
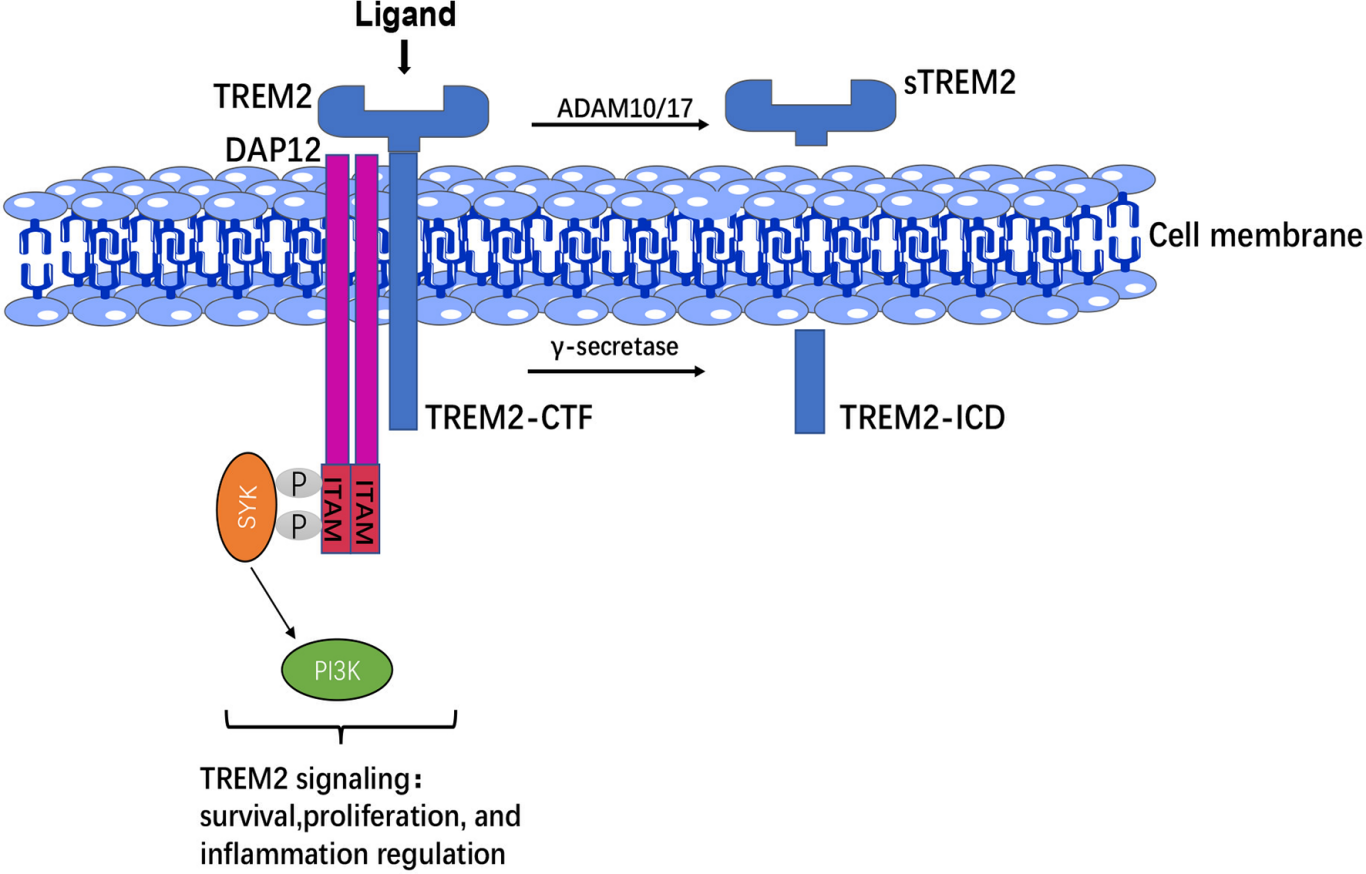
TREM2 structure and signaling. The extracellular domain of TREM2 was cleaved by ADAM 10/17 to produce sTREM2, whereas C-terminal fragment of TREM2 (TREM2-CTF) was cleaved by γ-secretase into intracellular domain (ICD). TREM2-DAP12 led to phosphorylation of ITAM, then recruitment of SYK to activate PI3K signaling and thus regulation of the survival, proliferation, and inflammatory responses of microglia.

TREM2 is expressed on the membrane surface of myeloid cells, whereas DAP12 requires receptor binding to transport to the cell surface, suggesting that a third protein may drive the binding of the TREM2-DAP12 complex and initiate TREM2-DAP12 signaling (31). In CNS, TREM2-DAP12 leads to phosphorylation of immunoreceptor tyrosine-based activation motif (ITAM), recruitment of spleen tyrosine kinase (SYK), which activates phosphoinositide 3-kinase (PI3K), calcium activation, integrin activation, cytoskeletal rearrangement, mammalian target of rapamycin (mTOR) and mitogen-activated protein kinase (MAPK) signaling activation (32), and thus regulating the survival, phagocytosis and activation of microglia (**Figure 1**).

## TREM2 and PD Pathology

TREM2 gene is located on human chromosome 6p21.1. Mutations in TREM2 occur more frequently in the coding sequence and found in the 3’ un-translated region (UTR), the upstream of the transcription start site (33–35). Numerous findings reported TREM2 (rs75932628, p.R47H) is associated with increased PD risk (14, 15). R47H is located on the protein surface and impairs TREM2 binding to lipids by altering the structure of the ligand-binding region (36, 37). In addition, R47H exhibited significant metabolic deficits and impaired precise regulation of TREM2 glycosylation (38, 39). On the other hand, the variation of R47H in the Northern European population was higher than that in the non-Northern European population. Recently, R47H was identified to increase PD susceptibility in American, Polish, Irish and Spanish population and it was unlikely to be a major genetic risk contributor of PD in Greek and Han Chinese population (14, 40, 41). Also, studies confirmed that R47H might increase the risk of PD in North Americans, but not in Europeans (7, 42). However, several studies reported that R47H might increase the risk of PD in European- descended populations (43). These controversial results might be due to the small gene frequency of the R47H polymorphism and the limited sample size of the case-control study experiment.

Additionally, further studies revealed that the biological consequences of TREM2 mutations could be mediated by inhibiting TREM2 receptor signaling or altering the production of other biologically active TREM2 cleavages, such as sTREM2. In addition, TREM2 variants do not affect TREM2 expression levels, suggesting that the effects of TREM2 variants occurred at the post-transcriptional level (44), which might be driven by post-transcription-related regulation. Due to the low mutation rate of TREM2, it is difficult to study in a well-matched population. Meanwhile, wild-type and mutant TREM2 are successfully produced based on expression systems in mammalian cells for structural and biophysical studies, which is critical for understanding the functional consequences of TREM2 mutations associated with the development of neurodegenerative diseases (45).

### TREM2 and Neuroinflammation

Autopsy and PET of PD patients exhibited the increased activation of microglia and expression of inflammatory factors on the damaged neurons in the substantia nigra of midbrain (46–48). Same results have been found in various PD animal models. More and more evidence suggests that microglia-mediated neuroinflammation is a key factor and significant feature in the pathogenesis of PD.

TREM2 is expressed only on the surface of microglia in CNS and modulates microglia-mediated neuroinflammation (49). Previous studies have confirmed that microglia have two phenotypes, pro-inflammatory phenotype (M1) and anti-inflammatory phenotype (M2) and TREM2 promotes the transformation of M1 microglia to M2 microglia. On the other hand, TREM2 has been found to be associated with the activation of disease-associated microglia (DAM) (50). Whether DAM are present in PD has not been verified. In MPTP-induced mouse PD models, TREM2 inhibited neuroinflammatory responses and reduced MPTP-induced neuropathic changes by inhibiting toll-like receptor 4(TLR4)/tumor necrosis factor receptor-associated factor 6 (TRAF6)-mediated nuclear factor-κB (NF-κB) and MAPK signaling activation (51). Also, TREM2 inhibited neuroinflammation by down-regulating PI3K/AKT and NF-κB signaling in lipopolysaccharide (LPS)-stimulated BV2 cells (52). Conditioned media from TREM2-siRNA transfected BV2 microglia induced apoptosis of cultured SH-SY5Y cells by inhibiting TREM2 expression and increasing pro-inflammatory factors expressions (53). Moreover, the expression of TREM2 was increased in the inflammatory state *in vivo*, but decreased *in vitro* by inflammatory stimulation (54). Likewise, although TREM2 function is classically described as promoting an anti-inflammatory phenotype, several lines of evidence demonstrated a pro-inflammatory role of TREM2 (35), suggesting that its role in inflammation is much more complex and these conflicting results might be related to changes in phenotypes and signaling pathway transduction based on different cell states and activities.

Furthermore, NLRP3 inflammasome in microglia recruited and activated caspase-1, which produced interleukin-1β (IL-1β) *via* shearing pro-IL-1β and then damaged DA neurons (55). Another evidence indicated that TLR4 mediated NLRP3 inflammasome activation in a NF-κB-dependent manner in CNS (56), whereas TREM2 negatively regulated TLR4-mediated NF-κB signaling pathway activation (51, 57), suggesting that TREM2 might inhibit neuroinflammation by down-regulating NLRP3 signaling activation. In turn, NF-κB signaling also down-regulated the expression of TREM2 (58). On the other hand, DA neurons produced TREM2 ligand (TREM2-L) (59). However, whether DA neurons activated TREM2 through TREM2-L had not been studied. In addition, binding of galactolectin 3(GAL3) to TLR4/TREM2 further activated microglia, and inhibition of GAL3 might be potential benefit for PD treatment (60). Other studies found that interleukin-3 (IL-3) regulated microglial immune responses and acted on downstream pathways in TREM2, and loss of TREM2 reduced the protective effect of IL-3 on neurons (61). Increased interleukin-10 (IL-10) altered TREM2 signaling (62), and TREM2 might act as a negative immunoregulatory molecule through Syk pathway in an IL-10-dependent manner (63). Long-term presence of inflammatory factors also decreased the expression of TREM2 (64), suggesting that TREM2 could interact with inflammatory factors to regulate the inflammatory response in CNS.

**Figure 2:**
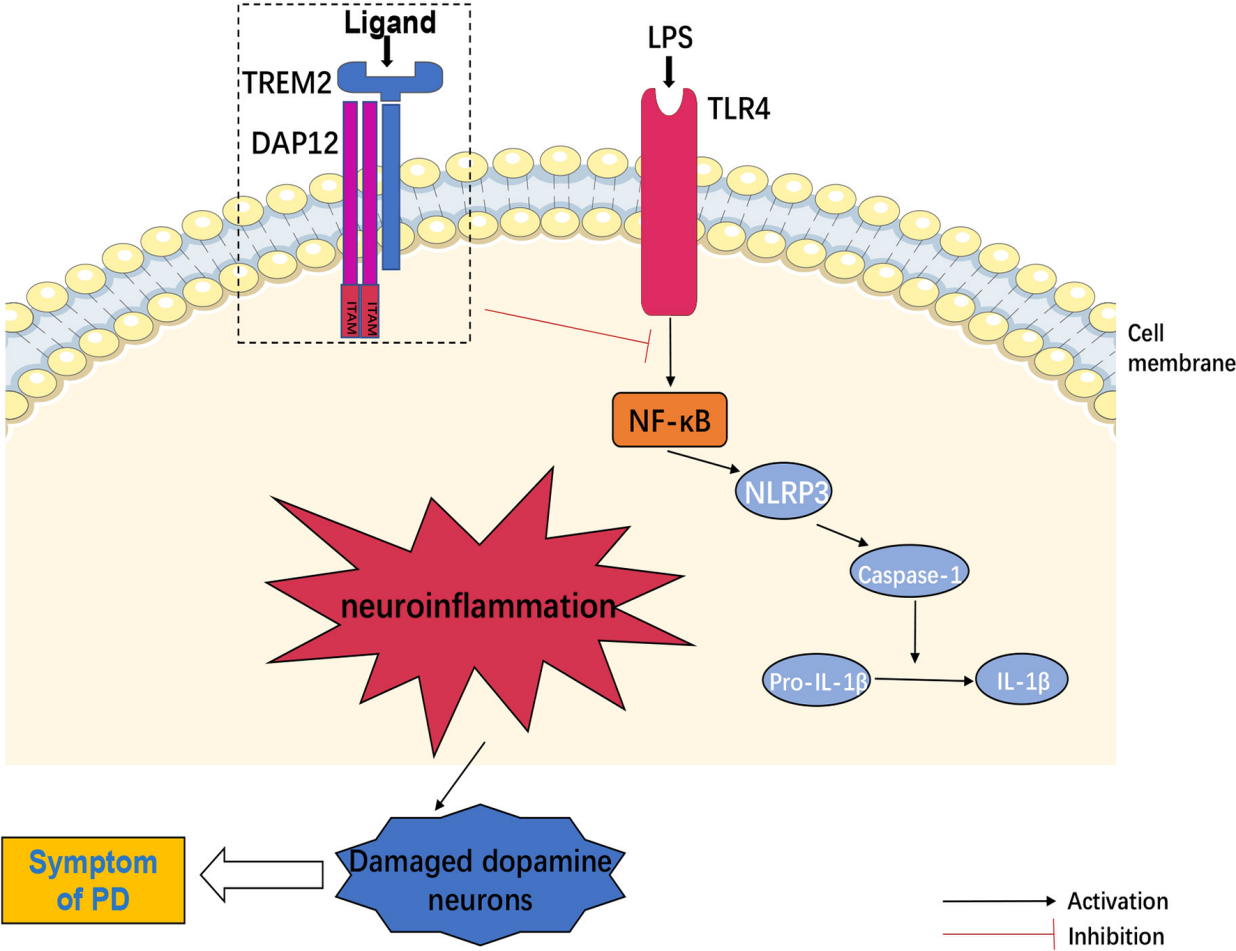
TREM2 and neuroinflammation. TLR4 mediated NLRP3 inflammasome activation in central nervous system (CNS) through regulation of NF-κB signaling pathway. NLRP3 inflammasome recruited and activated caspase-1, produced IL-1β by shearing pro-IL-1β and then induced neuroinflammation, which could damage dopamine neurons. However, TREM2 negatively regulated TLR4-mediated these neuroinflammatory signaling activation.

Meanwhile, TREM2/iNOS was a marker of increased anti-inflammatory factors, and melatonin could increase the proportion of TREM2/iNOS (65). Additionally, Nilotinib increased TREM2 levels in CSF and treated PD through its anti-inflammatory effects (66). It has also been found that pinitol increased the expression of TREM2 in BV2 microglia and inhibited the expression and secretion of pro-inflammatory cytokines, while silencing of TREM2 abolished the anti-inflammatory effects of pinitol (67). These findings suggested that therapeutic enhancement of TREM2 expression might be a new strategy for the intervention of neuroinflammation-induced PD. Moreover, sTREM2 was reflected in the activation of microglia during neuronal degeneration (68). sTREM2 stimulated the production of inflammatory cytokines through NF-κB (69). The reason might be sTREM2 competed with full-length TREM2 for ligand and inhibiting the anti-inflammatory effects of TREM2. Therefore, inhibition of sTREM2 signaling pathway might also be an effective therapeutic approach for PD (**Figure 2**).

### TREM2 and α-Syn

Aggregation of α -Syn monomer into amyloid fibrils through oligomeric intermediates was found to be the toxic mechanism leading to PD. Oligomers formed at the initial stage of aggregation were potent neurotoxic substances to cause cell death (70). TLR4 played the key role in the neurotoxicity induced by α-Syn oligomers (71). Infection of the midbrain with an adeno-associated virus vector overexpressing α -Syn and inoculation of preformed α -Syn fibrils into the striatum effectively induced DA neurodegeneration (72, 73). Compared with healthy controls, sTREM2 in CSF of PD patients was upregulated and positively correlated with total α-Syn degradation (74). Whether sTREM2 was related to α-Syn degradation remains to be studied. In addition, TREM2 mediated phagocytosis of microglia (75). α -Syn was be recognized by TREM2 on the cell membrane and then phagocytosed. Heat shock protein 60 (HSP60) in the chaperone binded to TREM2 and activated TREM2 and affected phagocytosis in TREM2 (76). During this process, beclin 1 played a critical role in regulating phagocytic receptor function in health and disease, and beclin-1 defects impaired TREM2 recycling (77). Thus, TREM2 might be closely associated with phagocytosis of α -Syn in PD but further studies are needed to determine whether α-Syn could be cleared after phagocytosis.

### TREM2 and Oxidative Stress

Oxidative stress was increased in CSF and blood of PD patients (78, 79), and oxidative stress was also found in animal models of PD (80). Oxidative stress products damaged DA neurons through DNA oxidation, protein oxidation and lipid peroxidation. Overexpression of TREM2 alleviated oxidative stress in hippocampal neurons by activating the PI3K/AKT signaling (81). Whether overexpression of TREM2 alleviated oxidative stress in DA neurons remains to be explored. MAPK is a key intermediate molecule in Mn-induced oxidative stress response, and dysregulated peroxisome proliferator-activated receptor γ (PPAR γ)/p38 MAPK signaling underlied the phenotypic deficits in TREM2 variants (38), and TREM2 variants enhanced oxidative stress in the CNS (82). DJ-1(PAPK7) is an oxidative stress sensor, and DJ-1 deficient microglia reduced the expression of TREM2 (83), suggesting that TREM2 was negatively associated with microglia oxidative stress.

Activated microglia are the main source of oxygen-free radical production, while mitochondrial dysfunction is due to disruption of the balance of reactive oxygen species (ROS) accumulation and utilization in cells and tissues. Overexpression of TREM2 inhibited mitochondrial ROS production in macrophages and thus NLRP3/caspase-1 inflammasome activation (84). R47H risk variant lead to mitochondrial dysfunction and stimulated inflammation through NLRP3-dependent inflammatory pathways (38, 85). TREM2 might inhibit NLRP3 activation and neuroinflammation by affecting ROS accumulation in microglia. In addition, TREM2 amplified ROS signaling and promoted osteoclastogenesis in periodontitis (86). Also, TREM2 regulated macrophage immune function by fine-tuning ROS to protect against bacterial infections (84). Oxidative stress was reduced in 24-months-old TREM2 knock-out mice (87). In AD mice, TREM2 activated a downstream pathway leading to mitochondrial damage (88). Thus, TREM2 may be associated differently with oxidative stress in different animal models, and its association with oxidative stress in PD models requires further investigation.

### TREM2 and Endoplasmic Reticulum Stress (ERS)

1-methyl-4-phenylpyridinium (MPP^+^) triggers the transient receptor potential vanilloid 4 (TRPV4) channel and induces ERS (89). Apoptosis induced by ERS overactivation is associated with the decreased DA neurons in PD patients (90). ERS is an abnormal state of lipid metabolism, and TREM2 is a lipid-lipoprotein-binding receptor (91). TREM2 mediated lipid metabolism in pluripotent stem cell-derived microglia-like cells through Phospholipase C gamma2 (PLCγ2) (92). Deficiency of TREM2 resulted in cholesterol ester (CE) overload and fewer lipid droplets, inducing ERS (93–95). ERS mediated changes in inflammatory and apoptotic pathways and was closely associated with cell growth, differentiation, survival and apoptosis. Lack of TREM2 could lead to microglial ERS, laying the foundation for subsequent neurodegenerative mechanisms. In the future, as the negative regulator, overexpression of TREM2 in human or animal models could be considered to observe whether ERS was alleviated.

### TREM2 and Iron-Induced Neurodegeneration

Iron-induced neurodegeneration is caused by the imbalance between the generation and degradation of intracellular lipid ROS. The iron deposition has been found in the substantia nigra of PD patients and animal models (96). The iron mediated α-Syn aggregation, oxidative stress and toxic effects on DA neurons (97, 98). The capacity of microglia to store iron was three times that of neurons (99), and microglia expressed transporters/molecules involved in brain iron metabolism, inducing ferritin synthesis (100). It has been found that iron overload increased the expression of TREM2 (101), and iron might be involved in various pathogenesis of PD by regulating TREM2 expression in microglia. Whether increased TREM2 expression caused by iron overload could affect ROS production, α-Syn phagocytosis and neuroinflammatory responses is poorly studied and warrants further exploration.

### TREM2 and Microbiota-Gut-Brain Axis

**Figure 3:**
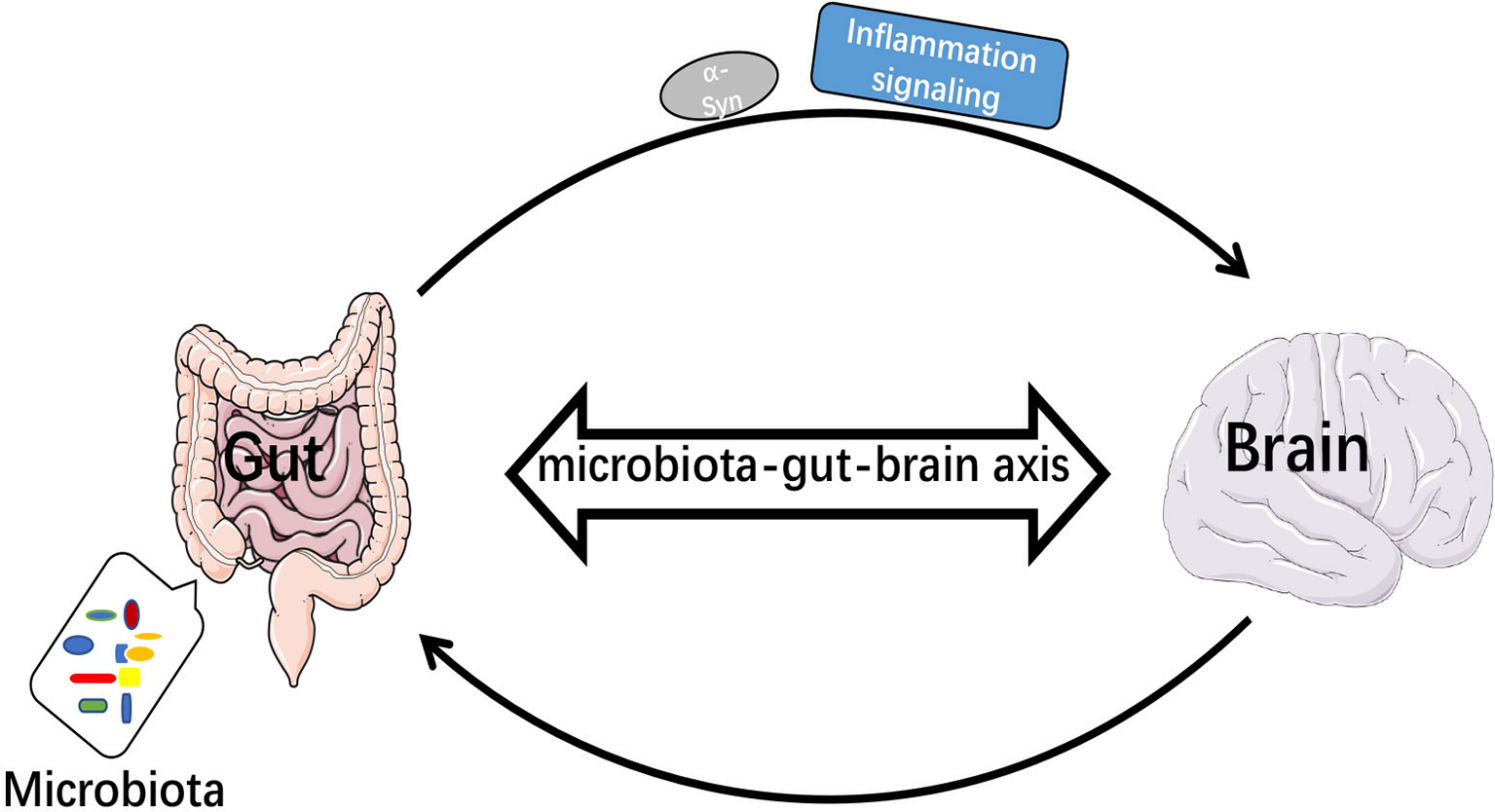
Microbiota-gut-brain axis transmitted inflammatory signals and α-Syn. When intestinal bacteria were imbalanced, inflammatory signals were transmitted to the brain *via* the bacteria-gut-brain axis and α-Syn was transmitted from enteric nervous system (ENS) to central nervous system (CNS).

A total of 80% of PD patients is accompanied by gastrointestinal (GI) dysfunction (102). The imbalance of gut microbiota leads to increased intestinal permeability, and inflammatory signals are transmitted to the brain through the gut-brain axis (103). Biopsies of GI tissue from both PD patients and healthy individuals revealed accumulation of α-Syn in the stomach, duodenum and colon (104–106), and α-Syn could be transferred from the enteric nervous system (ENS) to CNS *via* the vagus nerve (107). Impairment of intestinal barrier function or immune cell activation will promote Th1 cytokines that will overwhelm the ability of TREM2 and cause impaired healing (108). Intestinal dysbiosis may exacerbate neuroinflammation in brain through inhibiting the function of TREM2. It has also been found that LPS, gram-positive and -negative bacteria and fungi could upregulate the expression of TREM2 (109). TREM2 magnified mucosal inflammation during the development of colitis in mice (110). Thus, TREM2 might have different effects on peripheral and central inflammatory responses, affecting intracerebral function by upregulating intestinal inflammatory response (**Figure 3**).

### TREM2 and Non-Coding RNA (ncRNA)

In PD, ncRNA regulates the α-Syn expression and LB formation, mitochondrial dysfunction and apoptosis (111). TREM2 is associated with multiple ncRNAs. For example, the level of TREM2 protein was regulated by small nuclear RNA (snRNA) and Mir-665 (112, 113). miR-101a regulated microglia activation and immune responses *via* TREM2 (114). Epigenetic mechanisms of miRNA-34a-mediated down-regulation of TREM2 expression led to neurodegeneration (115, 116); Specifically, miR-3473b was involved in PD pathogenesis by inhibiting the expression of TREM2 (117). Whether ncRNA is involved in PD development through the regulation of TREM2 is unclear and needs further study. ncRNA are novel and challenging drug targets due to their abilities to affect the expression of other genes. However, it has been difficult to identify whether ncRNA specifically acted on TREM2.

### TREM2 and Exosomes

Exosomes are small vesicles about 30-150 nm in diameter secreted by living cells that deliver α-Syn to neurons. The delivered α-Syn induced more neurotoxic aggregation than free α-Syn oligomers. Microglial exosomes were capable of inducing nigrostriatal degeneration (118). Microglia can also transfer neuroinflammatory signals through exosomes (119). Exosomes upregulated TREM2 mRNA expression in LPS-induced THP-1 cells (120). Inflammatory stimuli may upregulated TREM2 expression through exosomes. Moreover, sTREM2 in brain parenchyma could be carried by a subset of microglia, macrophages, or exosomes (121). However, how exosomes regulated TREM2 signaling activation is worthy of further illumination.

### TREM2 and Neurotrophic Factors

In AD mouse models, up-regulation of TREM2 induced microglia to express brain-derived neurotrophic factor (BDNF) (122). Fasudil may target microglial phagocytosis by upregulating the TREM2/DAP12 pathway, with the increased expression of BDNF and glial cell-derived neurotrophic factor (GDNF) (123). Therefore, TREM2 might protect DA neurons by regulating microglia to release neurotrophic factors. However, direct delivery of exogenous BDNF to the brain of patients and enhancement of BDNF expression through gene therapy have not been successful (124). Thus, the possibility of enhancing BDNF expression through TREM2 to treat PD is another field of interest.

## TREM2 Treatment

Since sTREM2 expression in CSF was increased in PD patients and was positively correlated with total α-Syn in CSF (74), it was suggested that sTREM2 in CSF could be used as a substitute immune biomarker for PD neuron injury. Studies have shown that the misdiagnosis rate of PD was about 25% (125), and correct biomarkers were conducive to the prevention and diagnosis of PD. Moreover, antibody-based radioligands could be used as PET radioligands to track TREM2 changes *in vivo* and understand the dynamic changes of TREM2 in the PD progress (126). Increased expression of TREM2 reduced pathological microglial responses and neuropathological and behavioral deficits (127). Endogenous small-molecule inhibitors of TREM2 included phosphatidylethanolamine and phosphatidylserine *in vivo* (128). In a mouse PD model, TREM2 was also down-regulated by LPS/interleukin-34 (IL-34)/interferon-γ (129, 130). In addition, *in vitro* studies revealed that CELF2 was a novel splicing regulator in exon 3 of TREM2 that regulates species-specific splicing in TREM2 and reduces the expression of TREM2 protein (131), suggesting that inhibition of multiple substances could increase the expression of TREM2 and make it possible to treat PD by increasing the expression of TREM2. The anti-human TREM2 agonistic monoclonal antibody, AL002c, tempered microglial inflammatory response. It was safe and well tolerated in a first-in-human phase I clinical trial, and might be a promising candidate for PD treatment (132). Possible treatments also included agonists of TREM2 signaling *in vivo* that stabilized TREM2 on the cell surface and reduced its shedding (126). However, whether this affected the immune system, metabolism and fertility and intervention at which stage of PD progression would be beneficial is unclear (133). Crystal packing interfaces analysis using the maltose binding protein (MBP)-TREM2 immunoglobulin (Ig) fusion construct has shown that the surface of the TREM2 Ig domain can bind small molecules, providing potential utility for the discovery of new therapies for TREM2 (134).

## Conclusion

In recent years, the role of TREM2 in neurodegenerative diseases has rapidly become an interesting area of active studies. TREM2 adds to the understanding of DAM phenotypes. Currently, there is no effective drug treatment for PD, the reason might be that the etiology and mechanism of PD are still unclear and animal models cannot accurately represent the disease. Until now, there is an increasing focus on single-target therapy for PD. Although TREM2 could affect multiple pathogenesis of PD, including attenuating immune responses, α-Syn aggregation and oxidative stress, multi-target PD-related pathogenesis with TREM2 might be a promising therapeutic option. Importantly, there is a general lack of clinical validation of the correlation between TREM2 and various mechanisms of PD. Thus, further validation is needed in clinical practice. It is now necessary to clarify: 1) TREM2 is a PD susceptibility factor in which populations; 2) In what context does TREM2 function, whether it is affected by time, how does TREM2 function, which pathologies of PD are affected by its function, changes in expression, and signal transduction, and whether it affects multiple PD pathologies simultaneously; 3) ADAM10/17 is an abscission enzyme in TREM2 expression, and its inhibitor might enhance TREM2 expression but has multi-target effects. Could selective competitive inhibitors be found for shedding sites in TREM2? Or is there a viable way to activate TREM2 expression *in vivo* with fewer side effects, and whether it could be a viable basis for the prevention or treatment of PD. Collectively, a full understanding of role of TREM2 in PD is essential to provide additional insights into the underlying pathology of PD with the ultimate goal of developing new treatments strategies.

## Data Availability Statement

The original contributions presented in the study are included in the article/supplementary material. Further inquiries can be directed to the corresponding author.

## Author Contributions

All authors listed have made a substantial, direct, and intellectual contribution to the work, and approved it for publication.

## Funding

This study was supported by the National Natural Science Foundation of China (No. 82160690), Science and Technology Foundation of Guizhou Province (No. ZK[2021]-014), the foundation for High-level Innovative Talents of Guizhou Province (No. 20164027), the Foundation for Excellent Young Talents of Zunyi Medical University (No. 201603) and Collaborative Innovation Center of Chinese Ministry of Education (No. 2020-39).

## Conflict of Interest

The authors declare that the research was conducted in the absence of any commercial or financial relationships that could be construed as a potential conflict of interest.

## Publisher’s Note

All claims expressed in this article are solely those of the authors and do not necessarily represent those of their affiliated organizations, or those of the publisher, the editors and the reviewers. Any product that may be evaluated in this article, or claim that may be made by its manufacturer, is not guaranteed or endorsed by the publisher.
